# Evolution of Haemophilia Care in Europe: 10 years of the principles of care

**DOI:** 10.1186/s13023-020-01456-y

**Published:** 2020-07-13

**Authors:** D. Noone, B. O’Mahony, F. Peyvandi, M. Makris, A. Bok

**Affiliations:** 1 European Haemophilia Consortium, Brussels, Belgium; 2 Trinity College Dublin, Dublin, Ireland; 3 Angelo Bianchi Bonomi Hemophilia and Thrombosis Center and Fondazione Luigi Villa, Fondazione IRCCS Ca’ Granda Ospedale Maggiore Policlinico, Milan, Italy; 4 Department of Coagulation, Sheffield Haemophilia and Thrombosis Centre, Sheffield, UK

**Keywords:** Haemophilia, Healthcare management, Access

## Abstract

**Introduction:**

The European principles of care in haemophilia marked their first decade in 2018. These guiding principles were the beginning of the European Haemophilia Consortium (EHC) review of countries’ adherence to these principles in 2009, 2012, 2015 and 2018. The aim of this paper was to examine the implementation of the principles and how they have impacted the evolution of care in the last decade, as well as to identify remaining gaps and proposes future directions.

**Methods:**

In 2018, the EHC distributed a survey to EHC national member organisations in English and Russian and encouraged them to discuss responses with local clinicians for accuracy. Data was also cross-referenced and validated for countries in earlier surveys using additional available resources.

**Results:**

The 10-year-old European principles had a significant impact on the development of care for haemophilia and related bleeding disorders in Europe. They set objectives around which multi-stakeholder groups have established recommendations and specific steps for the progressive improvement of care for bleeding disorders. However, some have been promoted and implemented more than others.

**Conclusion:**

Monitoring adherence to, and impact of, the European Principles of Care significantly assists in tracking developments and highlighting gaps. Countries’ inability to report consistent and coherent data remains a challenge and hinders both provision of treatment and care for patients as well as optimal national and European healthcare systems.

## Introduction

In 2008, an interdisciplinary group of European clinicians with patient organisation input published 10 principles of haemophilia care that highlighted the importance of access to a multidisciplinary team of specialists in comprehensive care centres (CCC’s) and haemophilia treatment centres (HTCs), a supply of safe clotting factor concentrates for use in home treatment and prophylaxis programmes, and a national patient register collecting treatment statistics [1]. Since then, the European Haemophilia Consortium (EHC) has conducted regular surveys monitoring European adherence to the principles. Surveys conducted in 2009, 2012, 2015 and 2018 [2–4], revealed significant variation in the organisation of haemophilia care and access to factor concentrates across Europe. In 2013, Fischer et al., reported data from 21 individual centres from 14 countries, on the extent to which the principles of haemophilia care were being applied in these centres. That survey reported that the principles of care were generally well applied throughout those countries, however some aspects of centralisation, national organisation of care, use of registries, formal paediatric care and prophylaxis for adults could be improved [5]. In 2009, the ‘Wildbad Kreuth’ initiative which provides a platform for Council of Europe member states, including expert clinicians, healthcare workers and patient organisation representatives, created the first of a series of recommendations which were issued by the European Directorate for the Quality of Medicine and Healthcare (EDQM) [6]. Further recommendations from 2013 and 2016 were endorsed by the Council of Ministers of the Council of Europe which gives them significant influence in shaping policy nationally, have also been heavily guided by the orginal principles of care [7, 8]. EHC surveys tracked the implementation of the 10 principles of care and of EDQM recommendations with the results contributing to the EDQM discussions. Clinician-patient collaborations also established the European Haemophilia Network (EUHANET) and the European Haemophilia Safety Surveillance (EUHASS) system [9, 10]. This paper examines the implementation of the above principles and recommendations over 10 years, aims to identify remaining gaps and proposes future directions.

## Methods

In 2018, the EHC distributed a survey to the EHC network of national member organisations (NMOs). It was translated into English and Russian, which are the two languages used for data collection at national level for the EHC. Data was collected in collaboration with local clinicians and other agencies, such as academic institutions and health insurance funds. Responses were recorded through SurveyMonkey Inc. (San Mateo, California, USA). Paper responses were transposed into SurveyMonkey. Forty-two country responses were received which covers a total of 32,497 patients with haemophilia A and 6611 patients with haemophilia B. Four countries did not provide data on total patient numbers.

Datasets from 2009, 2012 and 2015 were combined with the most recent dataset in order to look at the impact of the principles in the last decade. As some of the initial datasets did not have complete data for all countries, other sources of data covering the same time frame were imputed, in order to expand the dataset and provide a more accurate depiction of the evolution of care within Europe. These secondary datasets included information from the World Federation of Hemophilia annual global surveys for 2008–2017 where data on patient numbers, clotting factor concentrate (CFC) use and percentage of patients on prophylaxis are reported for the same data collection timeframe [11]. Other public sources and published papers were also used, such as national data reporting CFC use on annual factor consumption within a calendar year [12, 13]. For the previous surveys, joint EHC and EAHAD certification of haemophilia centres through the EUHANET project was on-going. For the 2018 survey, data was compared to EUHANET reports and validated against centre certifications maps which reported CCCs and HTCs. Although no changes were made to the reporting of centres, this was an important step in understanding responses provided. For purposes of analysis, countries were divided into 3 regions which are primarily grouped by geography but also by historic access to haemophilia treatment:

- **Eastern: **Armenia, Azerbaijan, Belarus, Georgia, Kyrgyzstan, Moldova, Russia, Ukraine
- **Central: **Albania, Bosnia and Herzegovina, Bulgaria, Croatia, Czech Republic, Estonia, Greece, Hungary, Israel, Latvia, Lithuania, Macedonia, Montenegro, Poland, Romania, Serbia, Slovakia, Slovenia, Turkey
- **Western: **Austria, Belgium, Denmark, Finland, France, Germany, Iceland, Ireland, Italy, Netherlands, Norway, Portugal, Spain, Sweden, Switzerland, United Kingdom

Statistical analysis was performed with Stata version 13 (StataCorp, USA). No information was collected on education and research (Principle 10).

## Results

### Principle 1: establishment of a central haemophilia organisation

In 2018, 7 western, 9 central and 1 eastern European countries (50, 50 and 13% respectively) reported having this body (referred to as ‘National Haemophilia Council’ [NHC]). Data results varied over time; with some countries reporting an NHC in some years and in other years reporting an absence or inactivity of the NHC. Overall, there has been little consistent growth since 2009.

### Principle 2: national haemophilia patient registries

**Fig. 1:**
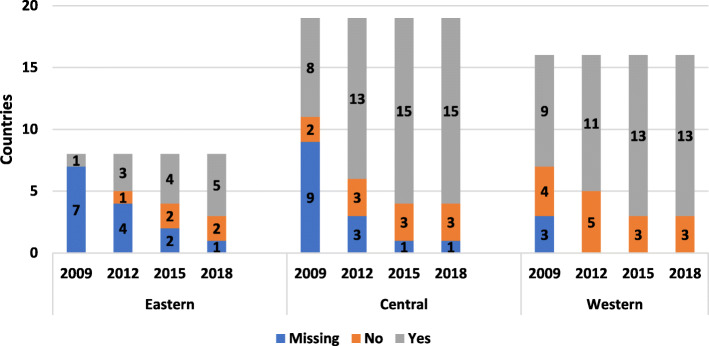
Countries with national registries

The development of registries in central and western Europe has steadily increased in the last decade (Fig. 1). In eastern Europe, there was an increase between 2015 and 2018. Belgium, Denmark and Portugal are the only countries currently without a national registry in western Europe. Denmark has children’s and adult registries that are not national. In central Europe, Bulgaria, Estonia and Lithuania have no national registries. In eastern Europe, Kyrgyzstan and Ukraine have no national registries. Each succeeding survey showed an increase in registry data being centrally coordinated (Supplementary Figure 1).

### Principle 3: a network of multidisciplinary comprehensive care and haemophilia treatment centres

In 2018, 12 western European countries reported having a haemophilia treatment classification structure; in central Europe 5 countries and in eastern Europe 1 country reported a classification (Supplementary Figure 2).

Historically most western countries have had access to CCCs with central and eastern European regions previously lacking access to CCCs (Supplementary Table 1). Since 2009 those regions have had steady growth in access to CCCs. The only western European country not to have a CCC is Iceland due to the survey definition requiring more than 40 severe haemophilia patients treated and the country currently reporting 20 severe patients. For western Europe, there was a slight reduction in HTC-defined centres since 2009. Norway and Sweden reported CCCs only for all 4 surveys. The Netherlands and Denmark reported CCCs in 2015 and 2018. In central Europe there was an increase in the number of HTCs being recognised as CCCs. Bosnia and Herzegovina, Slovenia, Poland and Israel reported CCCs for the first time. Estonia has no CCC. Montenegro has no defined CCC or HTC.

### Principle 4: partnership in the delivery of haemophilia care

Collaboration between patient representatives, clinicians and payers in decision-making regarding the delivery of haemophilia care has slightly increased in all European regions since 2009. In 2009, in eastern, central and western Europe respectively, 1, 3 and 5 countries (13, 16 and 32% respectively), reported that all three groups were represented and involved in decision-making on delivery of care. By 2018, 4 countries in eastern Europe, 7 countries in central Europe and 11 countries in western Europe (50, 37 and 69% respectively) reported involvement of all 3 groups in the decision-making on the delivery of care.

### Principle 5: safe and effective concentrates at optimum treatment levels

Reliance on whole plasma or cryoprecipitate has reduced in all regions except for occasional use where access to factor concentrates is limited. Based on volume usage there seems to be greater access to plasma-derived concentrates, especially in central and eastern Europe. In 2009, the mean European FVIII use was 3.42 IU/capita with vast access disparity ranging from 0.05 to 8.5 IU/capita across Europe [2]. By 2018, this mean rose to 5.48 (range 0.27–12.63).

**Fig. 2:**
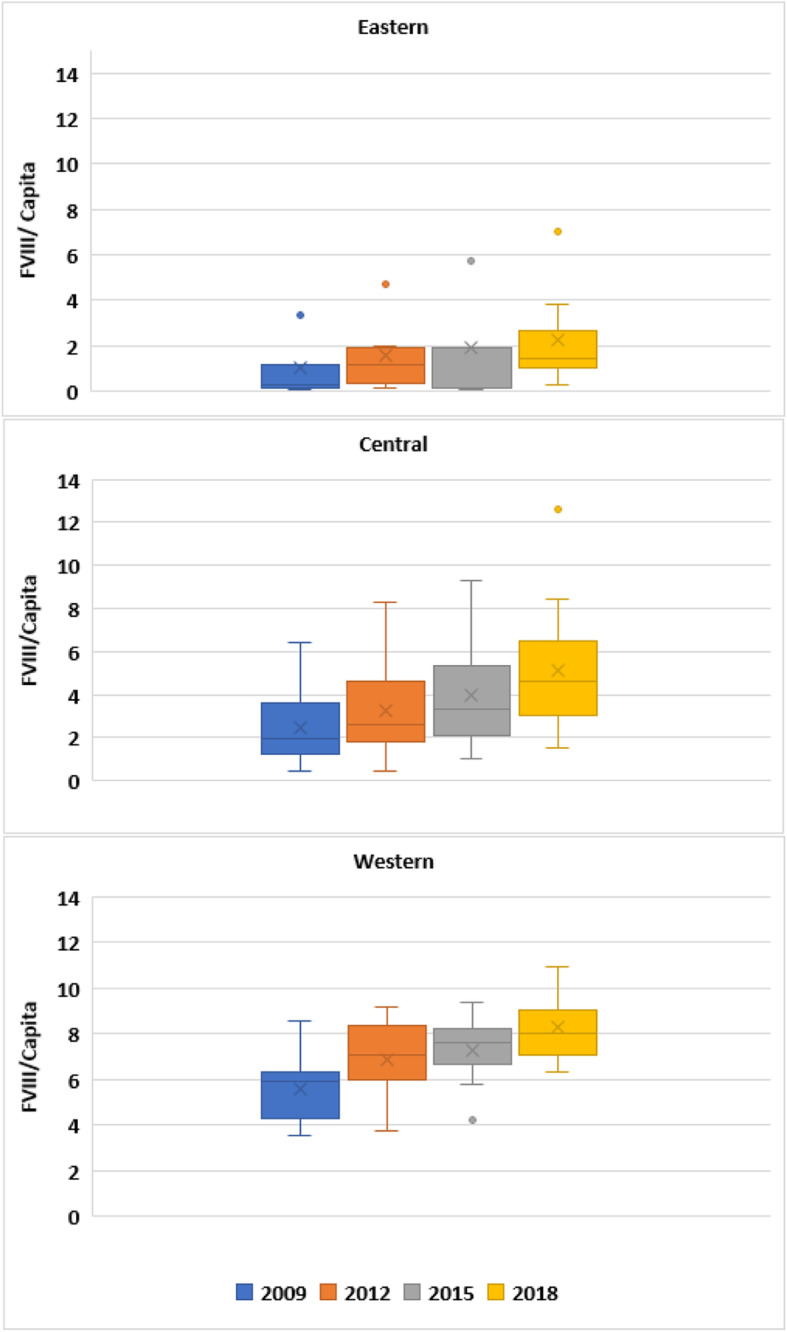
Change in FVIII/capita across eastern, central and western Europe from 2009 to 2018

In this timeframe central Europe increased from a mean of 2.5 to 5.1 IU/capita and eastern Europe from 1 IU to 2.24 IU/capita, although significant increases in Russia may skew this mean regional figure (Fig. 2). The growth in FIX has not been as significant with a mean of 0.61 IU/capita in 2009 and 0.77 IU/capita in 2018. As with FVIII, western Europe has shown the most growth from 2009 to 2018 (0.99 v 1.36 IU/capita) followed by central (0.40 v 0.58 /capita) and eastern Europe (0.26 v 0.26 IU/capita). In 2018, survey questions included access to extended half-life (EHL) products; 5 countries reported access to EHLs with the remainder reporting no or limited access.

**Fig. 3:**
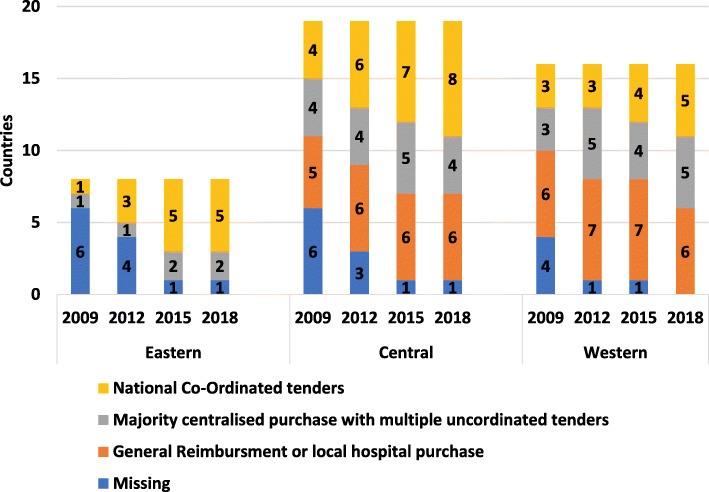
Variations in procurement of factor concentrates across Europe

Haemophilia is predominantly dosed based on weight; therefore, product procurement relies on relatively predictable national volumes. 2018 responses were coded into 3 categories: 1) countries whose haemophilia products were reimbursed by the general healthcare system or purchased on a hospital or insurance basis; 2) countries with multiple regional tenders or a majority-purchase through a centralized tender and a minority-purchase via uncoordinated, independent tenders, e.g. for adults/children, plasma-derived/recombinant, in-patient/home treatment or immune tolerance induction (ITI); 3) countries with centralised coordinated procurement processes and centralised budget (Fig. 3). In central and eastern Europe, centralised co-ordinated procurement models are the most common, potentially due to one main hospital/HTC having purchased treatments and as volumes increased, that centralised procurement process remained. In western Europe there has been an increase in countries working towards national tenders. Results show a positive correlation between increasing levels of coordination or national procurement leading to greater access to factor concentrates.

### Principle 6: home treatment and delivery

**Fig. 4:**
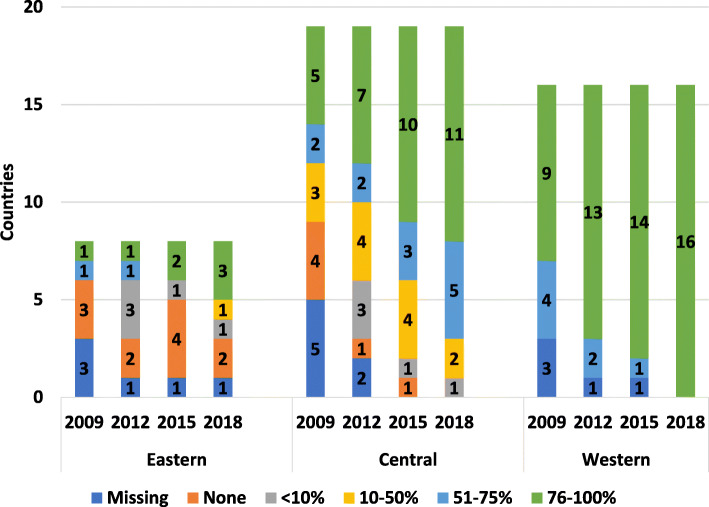
Patients within each country reported access to home treatment (%)

2018 results showed all patients in western countries requiring home treatment having access (Fig. 4). Central European patients saw significant growth in access to home treatment between 2009 to 2018, with more than half of the countries reporting 76–100% of patients requiring home treatment having access. Eastern Europe still lacks full access to home treatment for those requiring it, with slow growth between 2009 and 2015 and no increased access since then.

### Principle 7: prophylaxis

**Fig. 5:**
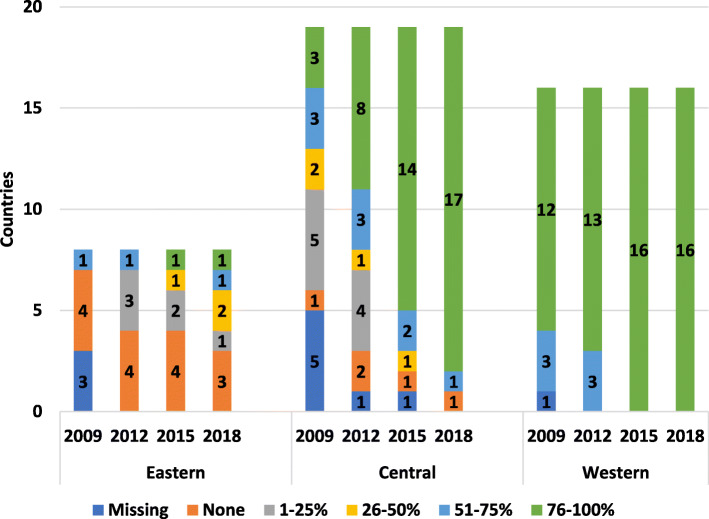
Availability of prophylaxis for children in countries

**Fig. 6:**
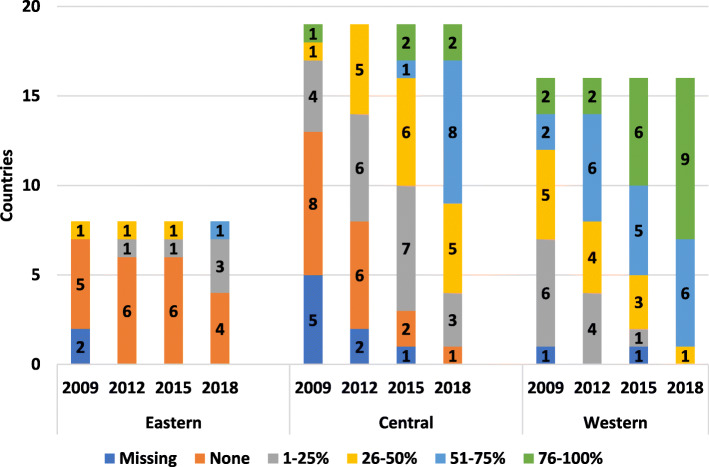
Availability of prophylaxis for adults in countries

In 2018 almost all western European children who required prophylaxis had access. Central Europe has shown significant progress since 2009; most children now have treatment to some form of prophylaxis, and this continues to increase (Fig. 5). Children in some eastern European countries still lack sufficient access. Most adults from western Europe avail of prophylactic regimens (Fig. 6) with a growing proportion in central and eastern Europe.

### Principle 8: specialist services and emergency care

NMOs were asked to rate access to specialist services within the country as a whole. There was a reported increase in access to emergency medicine and acute surgery without barriers from 37% of countries reporting in 2009 to 79% in 2018. Similar increases are seen in orthopedics (34 v 72%) and pediatrics (37 vs 86%). Reported access has also increased in other services like infectious disease specialists (35 v 63%), hepatology (32 v 60%), obstetrics and gynecology (25 v 60%), dental services (30 v 60%) and general surgery (35 v 63%). Significant access gaps persist in services such as physiotherapy (30 v 58%), genetic counselling (20 v 47%) and social and psychological support (19 v 33%), which have not grown to meet demand. An ageing population may increase, driving access to rheumatology (30 v 40%) and pain management (16 v 37%), but significant overall gaps in provision of care remain.

### Principle 9: management of inhibitors

**Fig. 7:**
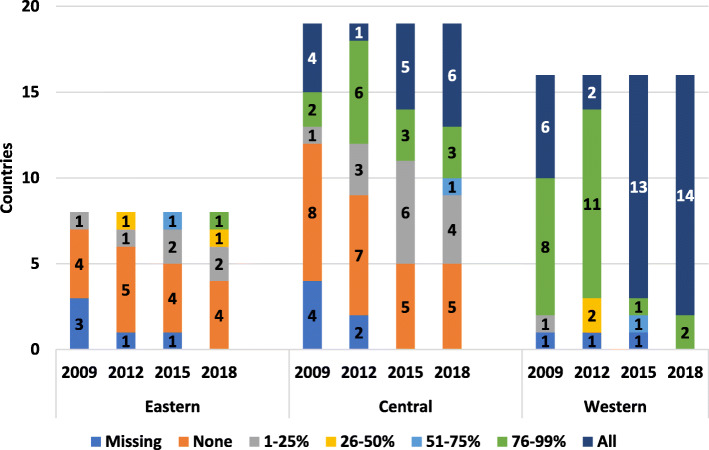
Reported access to Immune Tolerance Therapy (ITI) in Europe

**Fig. 8:**
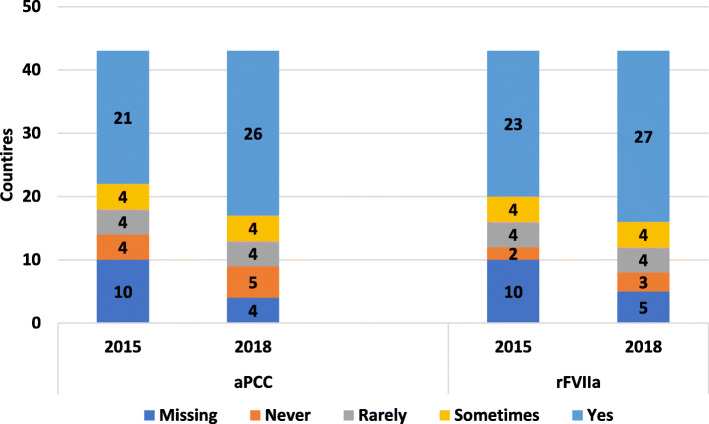
Access to by-passing agents in 2015 and 2018 reported across Europe

From 2009 to 2018, reported access to ITI increased in western European countries. Access in central European countries has also increased since 2009 but is not on par with western Europe (Fig. 7). In 2015 the survey included access to bypassing agents (BPA); while most countries report access, it is difficult for some countries to report on this consistently (Fig. 8).

### Principle 10: education and research

As the implementation of this important principle takes place more at European and cross-border levels than at national ones, it was never included in this survey work, although deserves a separate study to monitor its implementation.

## Discussion

The 10-year-old European principles of haemophilia care have had a significant impact on the development of care for haemophilia and related bleeding disorders in Europe. They set the objectives on which multi-stakeholder groups have established recommendations and specific steps for progressive improvement of care for bleeding disorders. However, some have been promoted and implemented more than others.

### Principle 1

The main function of a formal body like a NHC is to coordinate and ensure continued development of care for all bleeding disorders nationally. In countries lacking a NHC, data shows reduced strategic development of care. For example, results show slight increases in access to FIX treatment (but not to FVIII) in countries having a NHC, thus NHCs may act as levellers of care for smaller patient cohorts. One potential reason for countries reporting changing status of the NHC’s may be due to lack of legal status and statutory structures for governance of the committee.

### Principle 2

The importance of registries is generally understood and accepted since good data collection leads to maximal treatment benefits [14]. The presence of registries has increased since 2009. Results show that registries have greatest impact on care when centrally coordinated. For example, such registries provide more accurate information on variation in incidence and percentage of severe, moderate and mild patients; inhibitors; monitoring between sexes; and impact of inward/outward country migration. Centrally coordinated registries become even more important for future developments of care and safety monitoring with the European Medicines Agency (EMA) removing requirements for studies in previously untreated patients (PUPs), for ongoing discussions on inhibitors risks, and with the arrival of novel treatment products [15].

### Principle 3

A strong network of CCCs and HTCs ensures structure and strategic organisation and leads to the provision of optimum levels of treatment and care. The EUHANET accreditation of centres has significantly helped frame the understanding of care structures nationally. Increases in CCCs in central and eastern Europe have led to increased patient access to treatment and care. Reductions in HTCs in western Europe have been observed due to closure of very small centres. CCCs and HTCs will remain vital to the organisation of national care but safeguarding their roles will require concerted efforts by all stakeholders.

### Principle 4

Multiple stakeholder-partnership in national decision-making is the most efficient path to optimum delivery of care. Such partnerships have slowly increased in the last decade but inconsistently. There were minimal increases in patient-and/or clinician-involvement in haemophilia procurement, despite evidence highlighting the benefits of such practices [16]. Clearer guidance is needed on how formal joint decision-making or co-design bodies responsible for the development and delivery of care, and including patient representatives, clinicians, payers and government authorities, can work most effectively.

### Principle 5

Calling for safe and effective concentrates, applying a metric of national IU/per capita and recommending its incremental growth over time, has had obvious success. Central and eastern European countries show greater access to factor concentrates, while plasma and cryoprecipitate are used only sporadically. Predominately western European countries report significant usage of recombinant products, but data shows that plasma-derived products predominate in central and eastern Europe, where access to recombinant products is limited. The arrival of EHLs may further increase this eastern displacement and thereby increase availability of safe and effective products across regions. Results also show a trend towards increased access to treatment in countries with centralised procurement processes covering all regions, treatment needs and using a centralised budget.

### Principle 6

The principle of access to home treatment is amongst the most successful with year-on-year reports of increased access. Where home treatment is not yet fully available, this is most frequently due to insufficient treatment products although occasionally it is due to legal obstacles.

### Principle 7

Access to prophylaxis for children and adults has increased in all regions. In western and central Europe, this increase may be due to children previously on primary prophylaxis now continuing prophylaxis as adults, and greater access to secondary prophylaxis as adult treatment expectations increase. Eastern European countries still struggle to provide enough access to prophylaxis. In future, it will be necessary to define minimum trough levels and improve access to care for mild and moderate patients.

### Principle 8

Specialist services such as dentistry, physiotherapy, obstetrics and gynaecology, genetic counselling and psychosocial support should be mainstay components of all haemophilia centres, yet between 2009 and 2018 access to these services was inconsistent in all regions. In future better structures ensuring patient access to such services will be needed to ensure improvements in care.

### Principle 9

Clear gaps and inconsistent inhibitor treatment persist for patients across Europe. Over the next decade it will be important to monitor whether inhibitor rates remain the same or change with novel therapies, and to define optimum treatment levels with BPA and other agents.

## Conclusion

Monitoring adherence to, and the impact of, the European principles of care and the EDQM recommendations significantly assists in tracking developments and highlighting gaps. Countries’ inability to report consistent and coherent data remains a challenge and hinders both provision of treatment and care for patients as well as optimal national and European healthcare systems. Ensuring coherent, consistent data collection and national adherence to European principles and recommendations should be the next decade’s goal for all European countries. Institutions at national and European levels, including the newly established European Reference Networks (ERNs), can also play key roles in ensuring translation of these Principles into reality. Additional analysis is also encouraged at national levels to better understand national trends, challenges and gaps to assess which of principles had the greatest impact on improvement in care.

## Supplementary information

**Additional file 1: Supplementary Table 1.** Report of the presence of HTC’s and CCC’s across Europe.

**Additional file 2: Supplementary Figure 1.** Countries with a HTC/agency with the responsibility for the registry. **Supplementary Figure 2.** Has a system of classification for haemophilia treatment centres.

## Data Availability

The datasets during and/or analysed during the current study available from the corresponding author on reasonable request.

## Abbreviations

BPA: By-Passing Agents
CCC: Comprehensive Care Centres
CoE: Council of Europe
EDQM: European Directorate for the Quality of Medicine and Healthcare
EHC: European Haemophilia Consortium
EHL: Extended Half-Life
EMA: European Medicines Agency
EUHANET: European Haemophilia Network
EUHASS: European Haemophilia Safety Surveillance
FIX: Factor IX
FVIII: Factor VIII
HTC: Haemophilia Treatment Centres
ITI: Immune Tolerance Induction
IU: International Unit
NHC: National Haemophilia Council
NMO: National Member Organisations
PUPs: Previously Untreated Patients
