# Supplementary material for: Evolution of Haemophilia Care in Europe: 10 years of the principles of care
Source: Orphanet J Rare Dis. 2020 Jul 13;15:184. doi: 10.1186/s13023-020-01456-y (PMC7358930; doi:10.1186/s13023-020-01456-y)
Supplement: Supplementary file 1 — Additional file 1: Supplementary Table 1. Report of the presence of HTC’s and CCC’s across Europe. [file 13023_2020_1456_MOESM1_ESM.docx]

**Supplementary Tables**

Supplementary Table 1: Report of the presence of HTC's and CCC's across Europe

|  |  | **2009(n)** | **2012(n)** | **2015(n)** | **2018(n)** |
| --- | --- | --- | --- | --- | --- |
| **Comprehensive**  **Care**  **Centres (ccc)** | Eastern | 3 | 3 | 4 | 6 |
|  | Central | 11 | 9 | 14 | 16 |
|  | Western | 15 | 15 | 15 | 15 |
|  |  |  |  |  |  |
| **Haemophilia Treatment Centres (HTC)** | Eastern | 6 | 6 | 6 | 7 |
|  | Central | 15 | 15 | 16 | 14 |
|  | Western | 14 | 14 | 12 | 12 |
