# Supplementary material for: Evolution of Haemophilia Care in Europe: 10 years of the principles of care
Source: Orphanet J Rare Dis. 2020 Jul 13;15:184. doi: 10.1186/s13023-020-01456-y (PMC7358930; doi:10.1186/s13023-020-01456-y)
Supplement: Supplementary file 2 — Additional file 2: Supplementary Figure 1. Countries with a HTC/agency with the responsibility for the registry. Supplementary Figure 2. Has a system of classification for haemophilia treatment centres. [file 13023_2020_1456_MOESM2_ESM.docx]

**Supplementary Figures**

Supplementary Figure 1: Countries with a HTC/agency with the responsibility for the registry

Supplementary Figure 2: Has a system of classification for haemophilia treatment centres
